# The Aroma, Taste Contributions, and Flavor Evaluation Based on GC-IMS, E-Nose, and E-Tongue in Soybean Pastes: A Comparative Study

**DOI:** 10.3390/foods14071178

**Published:** 2025-03-27

**Authors:** Bing Yang, Heng Wang, Zhenxia Cao, Jing Yan, Zijie Dong, Fazheng Ren, Wanli Zhang, Lishui Chen

**Affiliations:** 1 Food Laboratory of Zhong Yuan, Luohe 462300, China; 2 Key Laboratory of Precision Nutrition and Food Quality, Department of Nutrition and Health, China Agricultural University, Beijing 100193, China

**Keywords:** soybean paste, taste profile, fuzzy mathematics, GC-IMS, PLS-DA

## Abstract

The objective of this study was to assess and compare the characteristics of different soybean pastes by using intelligent sensory analysis. In this study, color, flavor, texture, and taste were regarded as four factors affecting the sensory quality of soybean pastes and the sensory quality of four different soybean pastes was evaluated using fuzzy mathematics. The sensory evaluation scores of samples L, Z, and W were very similar and significantly higher than that of sample Y. Gas chromatography–ion mobility spectrometry (GC-IMS) detected 111 volatile flavor compounds, with acids, alcohol, and ketones having a significantly higher relative content than other compounds, indicating their vital role in the flavor formation process of soybean pastes. Furthermore, partial least squares discriminant analysis (PLS-DA) model analysis identified 41 marker compounds that could differentiate the four types of soybean pastes. The overall odor and flavor profile were detected by the E-nose and E-tongue. These fundamental results lay the groundwork for future research on the similarities and differences between the flavor characteristics of different brands of soybean paste.

## 1. Introduction

Soybean paste has a history of nearly 3000 years in China; it was created by working people by making full use of the role of microorganisms [1]. Soybean paste is an important condiment in traditional Chinese cuisine and an indispensable category of food in people’s daily meals [2]. It is the embodiment of human great creativity and food culture progress of the invention. Soybean paste is rich in protein, vitamins, and minerals, which are beneficial to human health. It undergoes fermentation, which converts protein macromolecules into amino acids and peptides that can be easily digested and absorbed by the body. At the same time, some special flavor substances produced by fermentation give soybean paste unique sensory characteristics. Soybean paste can be directly dipped to enhance the taste of dishes, reduce the use of salt, and enhance the taste and freshness. Soybean paste is not only a condiment but also a potentially functional food with reported antioxidant [3], anti-hypertensive [4], anticancer, and anti-microbial activities [5] and effects on anemia and the immune system [6].

Soybean paste products in different regions differ greatly in sensory quality and morphology because of the differences in region and production technology, such as Chinese soybean paste (Da-jiang) [7,8], soy sauce [9], sufu [10,11], Japanese miso [12,13], natto [14,15], Korean doenjang [16,17], and Indonesian tempeh [18]. The industrial fermented soybean paste koji is dominated by a single strain, with short fermentation time and high sauce production efficiency, but the flavor is relatively simple and lacks depth. Compared with industrial fermented soybean paste, the natural fermented soybean paste produces richer flavor.

Flavor is the key factor affecting the quality and consumer acceptance of fermented seasoning [19]. The flavor of soybean paste directly affects consumers’ sensory evaluation of the product. Due to the lack of effective technical means, previous studies on aromatic active compounds and consumer preferences were insufficient. At present, many intelligent sensory evaluation tools have been used to qualitatively analyze its smell and taste, such as E-nose and E-tongue. However, volatile flavor components in the samples could not be analyzed accurately [20]. The most recent studies in sensory evaluation methodologies show that sensory outcomes are influenced by a multitude of factors such as food characteristics and oral physiological parameters. Consequently, the integration of multiple approaches for analyzing food aroma has emerged as a prevailing trend.

GC-IMS can provide a comprehensive evaluation and analysis of VOCs. It can be directly injected without pretreatment, which is convenient, fast, efficient, and accurate. It can effectively make up for the limitations of gas chromatography–mass spectrometry (GC-MS) in the selective adsorption and detection of volatile trace substances [21,22]. However, the lack of a comprehensive database limits its quantitative analysis of samples to some extent [23]. Miao et al. [24] identified 91 and 70 VOCs, mainly including acids, aldehydes, esters, and alcohols, by HS-GC-IMS and GC-O-QTOF/MS, respectively. The integration of SPME-GC-IMS, E-nose, and E-tongue technology facilitates a comprehensive analysis of food flavor at both macroscopic and microscopic levels. This approach is currently the predominant method for detecting volatile flavor substances in food [25].

Although some studies on the flavor and physicochemical properties of soybean paste have been published, aroma active components of soybean paste have not been well reported before, and the correlation between aroma characteristics and consumer preferences has not been compared. In this paper, four soybean paste samples were selected, the volatile substances of different samples were compared by GC-IMS, E-nose, and E-tongue technology, and the flavor substances with greater contribution in soybean paste were further identified in combination with PLS-DA. The differences in volatile substances among commercial soybean paste were analyzed, so as to evaluate and improve soybean paste flavor substances and seek breakthroughs on the basis of traditional fermented sauce. It provided reference for subsequent enterprises to develop unique new sauce products.

## 2. Materials and Methods

### 2.1. Sample Preparation

Four available types of specialty soybean paste from Henan Province, in China marked as L, Z, W, and Y were selected. The products of L, Z, and W were purchased from market, and sample Y was taken from a local sauce factory, Luohe, Henan province, China.

### 2.2. Establishment of Fuzzy Mathematics Model

#### 2.2.1. Sensory Evaluation

The sensory evaluation used color, flavor, texture, and taste as indicators (Table 1). A panel of twenty volunteers, all with professional food industry background and no sensory deficiencies and in good health, was selected. No discussion was allowed during the evaluation process. The panel objectively evaluated and scored the samples.

#### 2.2.2. Determination of Sensory Factor Set and Comment Set

Comprehensive sensory evaluation using fuzzy mathematics involves applying the principles of fuzzy mathematics to the sensory assessment of products. This method is particularly well-suited for evaluating subjective and hard-to-quantify characteristics, thereby providing more accurate, objective, reasonable, and scientifically robust evaluation outcomes [26].

The soybean pastes L, Z, W, and Y represent samples labeled 1–4, respectively. The sensory factor set U = {color, flavor, texture, taste} = {U_1_, U_2_, U_3_, U_4_} was established using the respective states of the soybean pastes as evaluation factors. The sensory comment set V = {V_1_, V_2_, V_3_, V_4_} was established, where V_1_ = “excellent (90–100)”, V_2_ = “good (80–89)”, V_3_ = “fair (70–79)”, and V_4_ = “poor (60–69)”. The factor weights set X = {color, flavor, texture, taste} = {0.2, 0.3, 0.2, 0.3}.

#### 2.2.3. Determination of Fuzzy Matrix

The completed scoring tables were analyzed to determine the voting rate for each factor at every level relative to the total number of respondents. This information was then used to construct a fuzzy relation matrix.

The data in the table were divided by 20 to determine the membership R of the four factors of the sample. The membership matrix is obtained by arranging the factors in rows. According to the principle of fuzzy transformation, Y is regarded as a comprehensive evaluation set containing the products to be evaluated. A fuzzy relation evaluation set is obtained, Y = XR, where X is the weight set and R is the fuzzy matrix. Finally, the comprehensive score matrix T is introduced to process the fuzzy relation evaluation set Y. According to the characteristics of sensory evaluation, the evaluation level set is set as K = {k1, k2, k3, k4}, where the evaluation grade set was K = {90, 80, 70, 60}.

### 2.3. Analysis of Volatile Compounds by HS-GC-IMS

The volatile compounds of soybean paste were analyzed by HS-GC-IMS (FlavourSpec, G.A.S., Dortmund, Germany). A sample of 1 g was placed into a 20 mL headspace bottle and sealed. The test used an automated headspace sampling method with an injection volume of 500 µL, incubation time of 20 min, incubation temperature of 60 °C, injection needle temperature of 85 °C, and incubation speed of 500 r/min.

Using the MXT-WAX column (30 m × 0.53 mm × 1 µm), GC separation was performed at 60 °C. The programmed flow of the carrier gas (nitrogen, 99.999% purity) was set as follows; the initial flow rate was 2 mL/min, held for 2 min, then linearly increased to 10 mL/min within 8 min, and finally increased to 100 mL/min within 10 min and held for 40 min. The IMS system was coupled to GC and maintained at 45 °C. The speed of the drift gas (nitrogen, 99.999% purity) was kept at 75 mL/min.

Using n-ketone C_4_–C_9_ as reference, the retention index (RI) of each volatile organic compound was calculated. The retention index and drift times were searched and compared with IMS database to qualitatively analyze the target compounds.

### 2.4. Analysis of E-Nose

The 10 gas sensors in the E-nose device (PEN3, AIRSENSE Analytics GmbH, Germany) were sensitive to different aromas. The main information granted by each sensor is shown in Table 2. A measure of 10 g of sample was transferred into a 50 mL vial and kept for 30 min to achieve headspace gas equilibration. The primary parameters were set as follows: flow rate = 400 mL/min; measurement time = 80 s; and cleaning time = 80 s.

### 2.5. Analysis of E-Tongue

The E-tongue (SA402B, Insent Inc., Atsugi, Japan) adopts artificial lipid film sensor technology similar to the taste working principle of human tongue, which can objectively and digitally evaluate the basic taste sensory indicators such as the bitterness, astringency, sourness, saltiness, and umami of food, and can also analyze the aftertaste-bitterness, aftertaste-astringency, and richness.

The instrument was equipped with 5 sensors (C00, AE1, CA0, CT0, AAE) and 2 standard electrodes. Three measurement phases were performed as follows: sample detection (30 s), aftertaste detection (30 s), and washing (120 s).

### 2.6. Statistical Analysis

The statistical data were tested three times (n = 3), and the results were expressed as mean ± standard deviations (SD), with a significant level of *p* < 0.05. All data and significant difference analyses were conducted using a one-way analysis of variance through SPSS version 26.0 (SPSS Inc., Chicago, IL, USA). The characteristic fingerprint and the difference plots were generated by Gallery Plot and Reporter software (G.A.S., Dortmund, Germany). Origin 2018 (MicroCal, Northampton, MA, USA) and SIMCA 14.1 (MKS Umetrics, Umea, Sweden) were used for data processing and graphing.

## 3. Results and Discussion

### 3.1. Result of Sensory Evaluation

Based on Table 1, the R of fuzzy evaluation matrix was composed of the percentage of votes of the color, flavor, texture, taste of samples in the grade of “excellent”, “good”, “fair”, and “poor”. The distribution of votes for each index and level were plotted in a Table 3.

(1) $$R_{1}=\left|\begin{array}{cccc} 0.25 & 0.35 & 0.4 & 0\\ 0.7 & 0.3 & 0 & 0\\ 0.75 & 0.2 & 0.05 & 0\\ 0.85 & 0.1 & 0.05 & 0 \end{array}\right|$$

(2) $$R_{2}=\left|\begin{array}{cccc} 0.65 & 0.35 & 0 & 0\\ 0.35 & 0.4 & 0.25 & 0\\ 0.55 & 0.2 & 0.25 & 0\\ 0.55 & 0.35 & 0.1 & 0 \end{array}\right|$$

(3) $$R_{3}=\left|\begin{array}{cccc} 0.8 & 0.2 & 0 & 0\\ 0.4 & 0.35 & 0.25 & 0\\ 0.45 & 0.45 & 0.1 & 0\\ 0.3 & 0.5 & 0.2 & 0 \end{array}\right|$$

(4) $$R_{4}=\left|\begin{array}{cccc} 0.1 & 0.3 & 0.6 & 0\\ 0.15 & 0.2 & 0.4 & 0.25\\ 0.05 & 0.45 & 0.5 & 0\\ 0.2 & 0.5 & 0.3 & 0 \end{array}\right|$$

By utilizing matrix multiplication to calculate the grade membership degree for each sample evaluation set, where *Y* = *X × **R***, the sensory evaluation results of soybean pastes can be obtained as follows:

(5) $$Y_{1}\;=\;X\;\times R=\left|\begin{array}{cccc} 0.2 & 0.3 & 0.2 & 0.3 \end{array}\right|\times \left|\begin{array}{cccc} 0.25 & 0.35 & 0.4 & 0\\ 0.7 & 0.3 & 0 & 0\\ 0.75 & 0.2 & 0.05 & 0\\ 0.85 & 0.1 & 0.05 & 0 \end{array}\right|\;=\;\{0.665, 0.23, 0.105, 0\}$$

Similarly, the result of the remaining groups was calculated. **Y_2_** = {0.51, 0.335, 0.155, 0}, **Y_3_** = {0.46, 0.385, 0.155, 0}, *Y*_4_ = {0.135, 0.36, 0.43, 0.075}. The fuzzy comprehensive evaluation score was determined using the formula *T* = *Y × K*, where the evaluation set K = {90, 80, 70, 60} and Y_1_ = {0.665, 0.23, 0.105, 0}. The comprehensive score for the first combination was calculated as

(6) $$T_{1}\;=\;Y_{1}\times K=\left|\begin{array}{cccc} 0.665 & 0.23 & 0.105 & 0 \end{array}\right|\;\times \;\left|\begin{array}{c} 90\\ 80\\ 70\\ 60 \end{array}\right|=\;85.6$$

Similarly, *T*_2_ = 83.55, *T*_3_ = 83.05 and *T*_4_ = 75.55.

The calculation revealed that *T*_4_ had the lowest sensory score, significantly lower than the other samples. The sensory scores of the four samples, ranked from highest to lowest, were *T*_1_, *T*_2_, *T*_3_, and *T*_4_. Therefore, the sensory evaluation of the four samples can be summarized as L > Z > W > Y.

### 3.2. HS-GC-IMS Analysis

**Figure 1:**
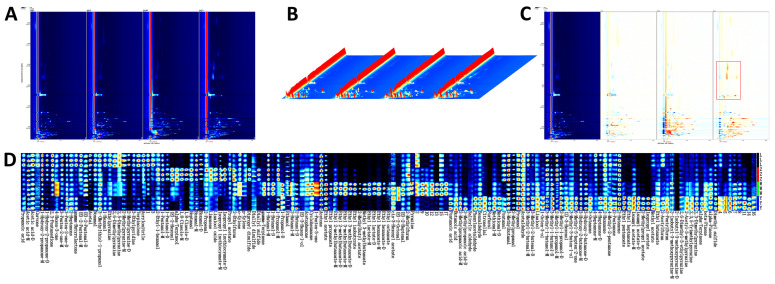
(**A**) 2D chromatograms of different soybean pastes in HS-GC-IMS. (**B**) 3D topographic plot of different soybean pastes in HS-GC-IMS. (**C**) Differential comparative chromatogram of different soybean pastes in HS-GC-IMS. (**D**) Fingerprints of different soybean pastes in HS-GC-IMS.

An important sensory characteristic that affects the preference and acceptance of soybean paste is its VOCs content, which varies from sample to sample [27]. The VOCs of different soybean paste were separated by GC-IMS, and the aroma compounds were clearly separated. The two-dimensional spectra of the aroma compounds of four different soybean pastes are shown in Figure 1A. In general, the deeper the concentration, the darker the color. The three axes of the three-dimensional spectrum (Figure 1B) represent migration time (X axis), retention time (Y axis), and signal peak intensity (Z axis). These axes allow for a clear visualization of the differences in VOCs across various samples. The standardized peak of active ions was indicated by a red vertical line on a blue background. Each point to the right of the ion peak (RIP) represents a VOC and the color difference represents the concentration of the substance, where high concentrations are in red and low concentrations in white [28]. To more clearly highlight the differences in VOCs between samples, we used the spectrum of L as a reference for comparison with other samples. A white background indicated consistency in detected VOCs, while red and blue background highlighted values higher and lower than the reference, respectively. As shown in Figure 1C, the VOCs of Y were obviously different from those of the other samples; specifically, the red area in Y was prominent, indicating that the compound content was obviously higher than that of the other samples.

**Figure 2:**
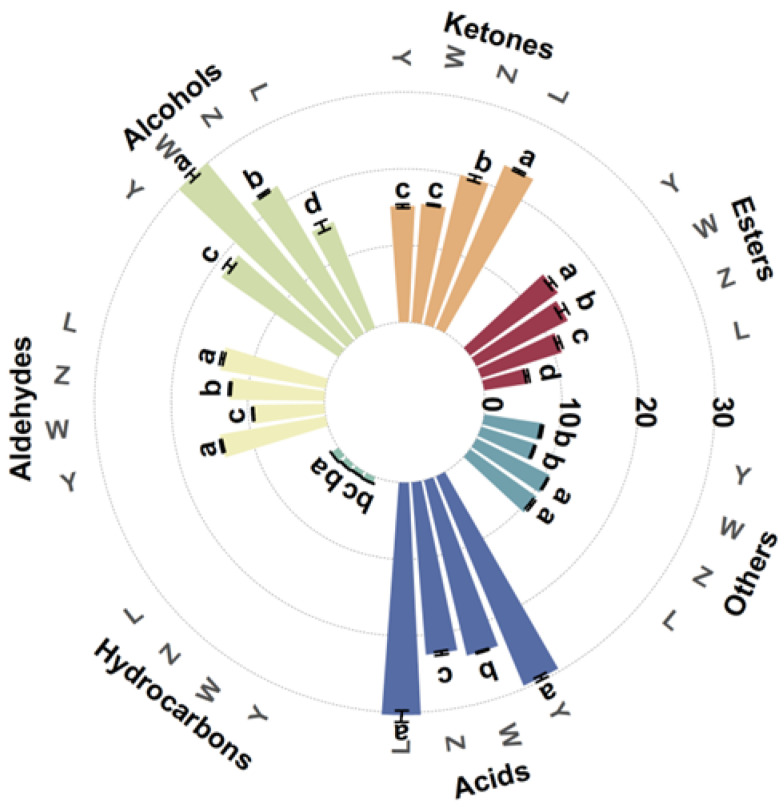
Relative content of VOCs of different soybean pastes. Different lower-case letters (a–d) indicate significant differences (*p* < 0.05).

Table 4 shows the mean relative area percentage and amount of each class of VOCs in various soybean paste samples, where a total of 111 VOCs, 136 peaks (including monomers and dimers) were detected. Figure 2 shows the average relative area percentage and amount of each class of volatile substances in the various soybean paste samples. These VOCs were divided into seven groups according to their chemical properties, including esters, ketones, alcohols, aldehydes, hydrocarbons, acids, and others. The results showed that the volatile flavor compounds of soybean pastes were mainly composed of acids, ketones, alcohols, and aldehydes, which accounted for 22.59–30.37%, 15.17–23.11%, 14.87–30.07%, and 9.38–14.04%, respectively. In L and Y, the relative percentage of acids was significantly higher than that of other compounds, while the relative percentage of alcohols in W was the highest, followed by acids. The proportion of alcohols, acids, and ketones in Z was similar. The hydrocarbon content in all samples was the lowest (0.76–1.17%). The types and contents of volatile compounds in the samples of different components were significantly different, but the influence of specific flavor compounds should be further analyzed in combination with the results of other detection indicators.

Propanoic acid, carvone, 1-hydroxy-2-propanone, 2,3-pentanedione, 4-hexen-3-one, gamma-butyrolactone, (E)-2-pentenal, acetal, ethylpyrazine, 2,5-dimethylpyrazine, 2-methylpyrazine, 3-ethylpyridine, terpinolene, and acrylonitrile were more abundant in sample L compared to others, and these compounds may originate from the oxidation of unsaturated fatty acids, the Maillard reaction, and the degradation of free amino acids. Ethanol, gamma-terpinene, 1-butanol, acetophenone, and acetone were presented in high levels in sample W. The sources of these compounds are generally divided into two pathways: microbial metabolism and non-enzymatic reactions [29].

Esters come from the reaction between acids and alcohols during the fermentation process and are important components in the flavor of soybean paste, and the samples of each soybean paste were rich in a variety of esters. Ethyl acetate content was the highest, creating a fresh, fruity, sweet, grassy taste [30].

Aldehydes and ketones in soybean paste are mainly produced by the degradation of amino acids and esters, which are important aroma components in fermented products, providing soybean paste with floral and burnt flavors. Ketones in L were significantly higher than those in Y (*p* < 0.05), but there was no significant difference in aldehydes (*p* > 0.05). The highest content of aldehydes was 3-methylbutanal, and the characteristic flavor was chocolate and fat. Furfural was significantly higher in Y than the other sample groups, with a creamy and bready flavor. Among the ketones, acetone and 1-hydroxy-2-propanone were high in content, and they, respectively, have a fresh, fruity aroma and a pungent odor and caramel taste.

Acids were relatively few in number but high in content. The acid mainly comes from the small molecular fatty acids produced during the process of fatty hydrolysis and oxidation, and its threshold is generally higher, and the contribution to the overall flavor is lower. As shown in Table 4, the relative content of acetic acid in L, Z, W, and Y was the highest. Zhang et al. [31] also confirmed by gas chromatography–mass spectrometry/olfactometry (GC–MS/O) that acetic acid is the key aroma compound of Yangjiang douchi (YD).

Alcohol compounds effectively promote the flavor formation of soybean paste. Ethanol, 2-methyl-1-propanol, 1-butanol, and 3-methyl-1-butanol were common alcohol compounds, among which 3-methyl-1-butanol was a branched alcohol separated from carbohydrates by the glycolytic pathway (EMP) during fermentation, giving it an aroma of whiskey and banana fruit.

Heterocyclic flavor substances in other samples were mostly derived from the Maillard reaction during the fermentation process and play an active role in developing soybean paste’s distinctive flavor. Pyrazine was essential for food flavor due to its low sensory threshold concentration. Pyrazines were significantly higher in L than in the other groups (*p* > 0.05), indicating richer nutty and roasted flavors. It was said that dimethyl sulfide was a unique flavor compound in many fermented foods, which acts as an aromatic compound and precursor in the reaction of producing more complex aromatic compounds [24]. Hydrocarbon compounds contribute little to flavor formation.

During the fermentation of soybean paste, protein was hydrolyzed by microorganisms, leading to an accumulation of free amino acids. These amino acids were the precursors of flavor substances, which were converted into pyrazines, pyrrole, and furans by the Maillard reaction, and converted into aldehydes and ketones by Stryker degradation, thus forming the characteristic flavor, aroma, and taste of soybean paste.

### 3.3. PLS-DA and Model Evaluation Analysis

**Figure 3:**
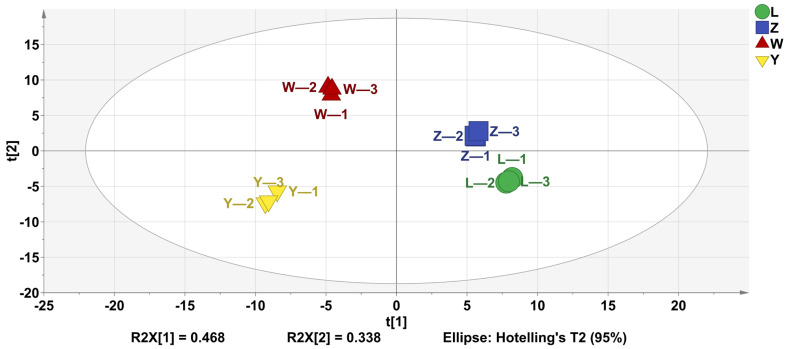
PLS-DA analysis of VOCs of different soybean pastes.

PLS-DA is widely utilized to analyze the volatile components of samples, reflecting the differences in volatile substances across different samples. As shown in Figure 3, the four groups achieved a good separation.

The analysis revealed that the independent variable fitting index (R^2^ X) was 0.946, the dependent variable fitting index (R^2^ Y) was 0.997, and the model prediction index (Q^2^) was 0.993. Given that both R^2^ and Q^2^ exceed 0.5, these values indicated that the model fit was robust and reliable.

These results demonstrated a strong model fit and satisfactory predictive accuracy. Therefore, PLS-DA can effectively differentiate the odor profiles of the four soybean pastes.

### 3.4. Key Volatile Substances

Variable Importance in Projection (VIP) is an important index to evaluate the importance of variables in distinguishing samples [32]. After detecting odorant compounds in the samples using GC-IMS, the VIP values obtained through a reliable PLS-DA model were used to evaluate the contribution of each odor compound to the overall aroma of different soybean pastes.

**Figure 4:**
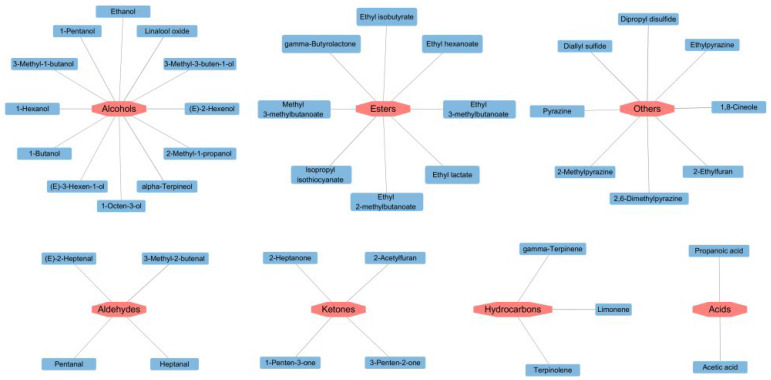
Volatile compounds with VIP > 1 of different soybean pastes.

As illustrated in Figure 4, key volatile substances screened by PLS-DA significantly contributed to the flavor of different soybean pastes. Compounds with VIP values > 1 were deemed characteristic aroma compounds, and higher VIP values indicated more substantial contributions to flavor differentiation. In this model, a total of 41 variables with VIP values > 1 were identified, namely 1,8-cineole, dipropyl disulfide, linalool oxide, limonene, diallyl sulfide, isopropyl isothiocyanate, alpha-terpineol, (E)-2-hexenol, 3-methyl-2-butenal, 1-hexanol, 2-ethylfuran, 2,6-dimethylpyrazine, acetic acid, 1-penten-3-one, 1-pentanol, gamma-butyrolactone, 2-acetylfuran, gamma-terpinene, propanoic acid, ethylpyrazine, ethyl lactate, ethyl hexanoate, methyl 3-methylbutanoate, 2-methylpyrazine, 2-methyl-1-propanol, 3-methyl-3-buten-1-ol, 3-penten-2-one, ethyl 3-methylbutanoate, (E)-2-heptenal, 2-heptanone, 1-octen-3-ol, terpinolene, heptanal, pyrazine, ethyl isobutyrate, 1-butanol, (E)-3-hexen-1-ol, ethyl 2-methylbutanoate, pentanal, ethanol, and 3-methyl-1-butanol. These compounds emerge as key differentiators for PLS-DA models for the HS-GC-IMS datasets.

### 3.5. E-Nose Analysis

**Figure 5:**
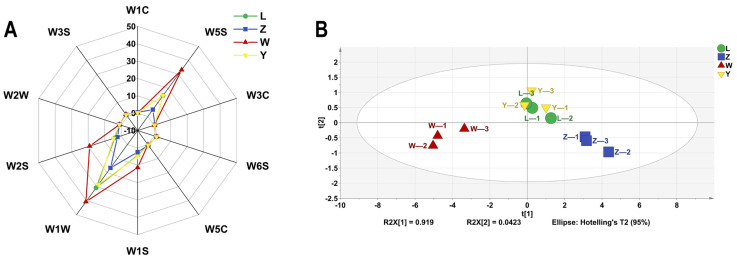
The VOCs of different soybean pastes based on E-nose data. (**A**) Radar chart and (**B**) PCA scores.

The E-nose system uses a range of sensors and pattern recognition technologies to measure and characterize volatile aroma compounds with high sensitivity, repeatability, and reliability [33]. As shown in Figure 5A, the sensors W1C, W3C, and W5C had almost no response to the soybean paste samples, indicating that the product contained very few aromatic compounds, ammonia compounds, and aromatic and short-chain alkanes. The W6S, W2W, and W3S sensors have lower signal intensities for the samples, indicating small amounts of hydrides, aromatic components, organic sulfides, and long-chain alkanes. The response values of soybean paste samples to W1S, W1W, and W2S were high, and there were large differences (*p* < 0.05) among samples. These three sensors can distinguish different brands of soybean paste samples.

According to the data shown in Figure 5B, it can be concluded that the first and second principal components account for 96.1% of the variance in the PCA analysis. This indicates that the main flavor characteristics of the sample have been accurately characterized, indicating the reliability and consistency of the sample. PCA results displayed that the samples were roughly divided into three relatively independent regions. The samples in the two periods of L and Y were more concentrated and those in Z and W were relatively independent. In terms of aroma, samples L and Y are very similar, with their horizontal coordinate distribution being closely aligned, indicating no significant difference between the two brands (*p* > 0.05). However, there is a marked change in the odor signature of the Z and W samples, as evidenced by the difference in their cross coordinates. Therefore, it is necessary to further analyze the effects of specific flavor compounds in combination with the results of GC-IMS.

### 3.6. E-Tongue Analysis

**Figure 6:**
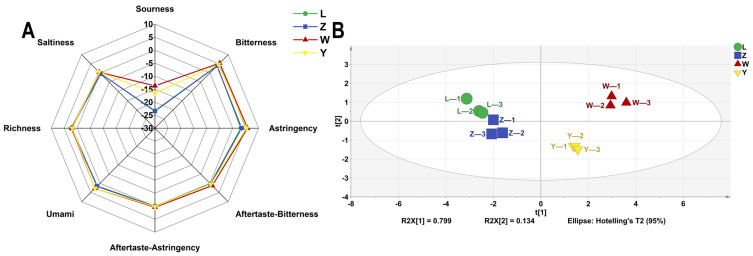
The VOCs of different soybean pastes based on E-tongue data. (**A**) Radar chart and (**B**) PCA scores.

The E-tongue system, which consists of sensors and auxiliary electronic components, can mimic the human taste organ and has the advantages of being non-destructive, efficient, and capable of online measurement and analysis. The outputs of the five sensors of the electronic tongue were plotted as a radar plot (Figure 6A). It revealed that sourness was below threshold of taste perception that and saltiness, aftertaste-astringency, and aftertaste-bitterness approached zero. Samples L and Z were similar but had lower sourness intensity and higher astringency and umami than samples W and Y. Electronic tongue testing was implemented to evaluate the taste profile of the samples, and a PCA plot with different soybean pastes is presented in Figure 6B. The PCA results clearly indicate that sample W is distinctly different from the other samples (L, Z, and Y), demonstrating significant independence. In contrast, samples L and Z exhibit closer similarities, suggesting only minor variations between them. The PC1 and PC2 accounted for 79.9% and 13.4%, respectively, with a combined total exceeding 85%. This indicates that the taste variations among the samples can be accurately captured and represented. The analysis distinctly reveals the differences in taste profiles among the four samples.

### 3.7. Correlation Analysis

#### 3.7.1. Correlation Between E-Nose and GC–IMS

**Figure 7:**
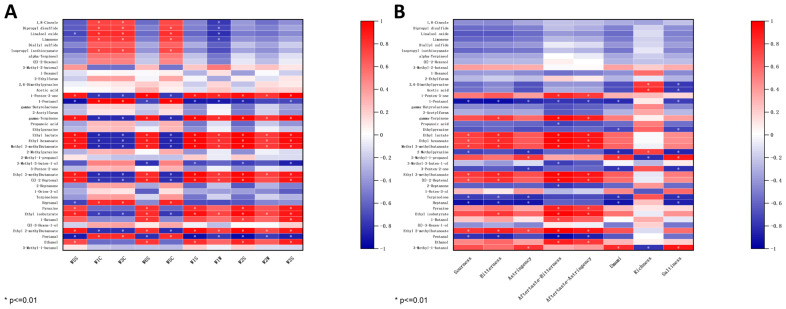
(**A**) Heatmap of the correlation between the response values of the E-nose sensors and the levels of differential volatile compounds. (**B**) Heatmap of the correlation between the response values of the E-tongue sensors and the levels of differential volatile compounds.

To enhance the overall efficiency of the E-nose and GC-IMS, a study was conducted to investigate the potential correlation between the E-nose sensor responses and the levels of volatile compounds detected by GC-IMS. As shown in Figure 7A, the analysis focused on the potential correlation between the response values of the E-nose sensor and the levels of 41 differential volatile compounds with VIP values > 1 detected by GC-IMS. Several sensors including W5S, W6S, W1S, W1W, W2S, W2W, and W3S showed a positive correlation with major compounds such as 1-Penten-3-one, gamma-Terpinene, Ethyl lactate, Ethyl hexanoate, Methyl 3-methylbutanoate, Ethyl 3-methylbutanoate, (E)-2-Heptenal, Ethyl isobutyrate, and Ethyl 2-methylbutaonate, which were identified at high levels in W compared to other samples through GC-IMS analysis. Conversely, sensors W1C, W3C, and W5C were negatively correlated with the above compounds. The remaining volatile compounds that did not show significant correlation (*p* > 0.01) with the E-nose sensor. Therefore, E-nose and GC-IMS can be used to identify four kinds of soybean pastes according to olfaction.

#### 3.7.2. Correlation Between E-Tongue and GC–IMS

The E-tongue can provide information on eight taste intensities of soybean pastes: sourness, bitterness, astringency, aftertaste-bitterness, aftertaste-astringency, umami, richness, saltiness. Consequently, the sensor response values from the E-tongue are correlated with the content levels of these eight flavor profiles. As shown in Figure 7B, sourness, bitterness, aftertaste-bitterness, and aftertaste-astringency were positively correlated with Ethyl lactate, Ethyl hexanoate, Methyl 3-methylbutanoate, Ethyl 3-methylbutanoate, (E)-2-Heptenal, and Ethyl 2-methylbutaonate, which were identified at high levels in W compared to other samples through GC-IMS analysis. Notably, umami and saltiness exhibited a negative correlation with 1-Pentanol, Ethylpyrazine, 2-Methylpyrazine, 3-Penten-2-one, Terpinolene, and Heptanal. The results indicated a certain correlation between the VOC content and the E-tongue sensor response values, suggesting that these two factors can be combined to effectively analyze the taste characteristics of soybean pastes.

## 4. Conclusions

This study reported the VOCs in four types of soybean pastes from Henan Province. Fuzzy mathematics was employed to comprehensively evaluate the characteristics of various soybean pastes. Based on the modeling and data output, the samples at were ranked as L > Z > W > Y. Sample Y had the lowest sensory score, which was significantly different from other samples (*p* < 0.05), but there was no significant difference among the other three samples (*p* > 0.05). A total of 111 volatile organic compounds were identified by GC-IMS, among which the relative contents of acids, alcohol, and ketones were higher. During the study, 41 compounds (VIP > 1) analyzed using the PLS-DA model, revealing that the combination of E-nose with GC-IMS and E-tongue with GC-IMS could effectively differentiate between four soybean pastes via their respective smells and tastes. The results of E-nose and E-tongue showed that the smell and taste of sample W was significantly different from the rest of the samples (L, Z, and Y). Overall, HS-GC-IMS, E-nose, and E-tongue were effective methods for detecting the flavor of soybean pastes, and further revealed the differences in the flavor of different soybean pastes, and provided a certain reference value for improving the quality and consumer acceptance of soybean pastes. Based on these findings, further exploration of the quality change rule and quality control mechanism in different processing stages is suggested in order to accelerate the production and development of soybean products.

## Figures and Tables

**Table 1:** Sensory evaluation standard of different soybean pastes.

Items	V_1_	V_2_	V_3_	V_4_
Color	Reddish-brown, bright, and shiny.	Reddish brown, shiny.	Dull surface color and low gloss.	The surface is not shiny, the color is gray and dull.
Flavor	Harmonious smell and strong sauce flavor.	Harmonious smell and sauce flavor.	Light sauce flavor, no bad flavor.	Light sauce flavor, peculiar flavor.
Texture	The sauce is medium consistency, uniform, no impurities.	The sauce is medium consistency, no impurities.	Paste consistency is not appropriate, no impurities.	Paste consistency is not appropriate and there are impurities.
Taste	Full taste, and mellow, and appropriate salinity.	Umami and appropriate salinity.	Slightly umami, slightly salty or light.	Burnt, too salty or too light.

Note: V_1_ = excellent (90–100); V_2_ = good (80–89); V_3_ = fair (70–79); and V_4_ = poor (60–69).

**Table 2:** Performance description of the E-nose sensors.

Sensor Number	Sensors Name	Sensitive Substance
1	W1C	Aromatic compounds
2	W5S	Nitrogen oxides
3	W3C	Ammonia, aromatic molecule
4	W6S	Hydrides
5	W5C	Olefin, aromatic components, and polar molecules
6	W1S	Alkanes
7	W1W	Sulfides
8	W2S	Alcohols and some aromatic compounds
9	W2W	Aromatic components and organic sulfides
10	W3S	Aliphatic series

**Table 3:** Statistics of sensory scores of evaluation indicators of different soybean pastes.

Sample	Color				Flavor				Texture				Taste			
	V_1_	V_2_	V_3_	V_4_	V_1_	V_2_	V_3_	V_4_	V_1_	V_2_	V_3_	V_4_	V_1_	V_2_	V_3_	V_4_
1	5	7	8	0	14	6	0	0	15	4	1	0	17	2	1	0
2	13	7	0	0	7	8	5	0	11	4	5	0	11	7	2	0
3	16	4	0	0	8	7	5	0	9	9	2	0	6	10	4	0
4	2	6	12	0	3	4	8	5	1	9	10	0	4	10	6	0

**Table 4:** The results of the determination of volatile substances of different soybean pastes in by GC-IMS.

Compounds (111)	MW	RI	Rt (s)	Dt (ms)	Relative Amount (%)			
					L	Z	W	Y
Esters (21)								
Methyl acetate	74.1	851.8	256.877	1.19121	0.32 ± 0.022 ^b^	0.14 ± 0.008 ^c^	0.12 ± 0.005 ^c^	0.46 ± 0.022 ^a^
Ethyl acetate	88.1	896.8	283.395	1.33437	1.31 ± 0.152 ^d^	1.88 ± 0.100 ^c^	4.07 ± 0.061 ^a^	3.30 ± 0.450 ^b^
Isopropyl acetate	102.1	957.6	323.64	1.47496	0.14 ± 0.028 ^b^	0.05 ± 0.001 ^b^	0.02 ± 0.003 ^b^	1.87 ± 0.140 ^a^
Ethyl propanoate	102.1	974.5	335.737	1.44596	0.03 ± 0.001 ^c^	0.05 ± 0.004 ^c^	0.23 ± 0.032 ^a^	0.09 ± 0.012 ^b^
Ethyl isobutyrate	116.2	982.3	341.553	1.55668	0.02 ± 0.001 ^c^	0.27 ± 0.038 ^b^	1.46 ± 0.156 ^a^	0.07 ± 0.010 ^c^
Propyl acetate	102.1	995.1	351.196	1.47584	0.21 ± 0.890 ^ab^	0.28 ± 0.103 ^a^	0.18 ± 0.007 ^ab^	0.12 ± 0.018 ^b^
2-Methylpropyl acetate	116.2	1030.6	388.863	1.60889	0.02 ± 0.000 ^b^	0.03 ± 0.004 ^b^	0.07 ± 0.014 ^b^	0.70 ± 0.084 ^a^
Ethyl butanoate	116.2	1038.3	397.602	1.19963	0.10 ± 0.003 ^c^	0.14 ± 0.009 ^b^	0.18 ± 0.012 ^a^	0.18 ± 0.008 ^a^
Methyl 3-methylbutanoate	116.2	1054.2	416.278	1.55364	0.01 ± 0.000 ^b^	0.01 ± 0.001 ^b^	0.17 ± 0.032 ^a^	0.02 ± 0.000 ^b^
Ethyl 2-methylbutanoate-M	130.2	1069.5	434.968	1.25521	0.05 ± 0.001 ^d^	0.15 ± 0.013 ^c^	0.32 ± 0.011 ^a^	0.18 ± 0.005 ^b^
Ethyl 2-methylbutanoate-D	130.2	1069.5	434.968	1.25521	0.02 ± 0.003 ^b^	0.04 ± 0.008 ^b^	0.65 ± 0.096 ^a^	0.04 ± 0.003 ^b^
Ethyl 3-methylbutanoate-M	130.2	1082.4	451.541	1.27458	0.02 ± 0.002 ^c^	0.09 ± 0.008 ^b^	0.50 ± 0.037 ^a^	0.09 ± 0.008 ^b^
Ethyl 3-methylbutanoate-D	130.2	1082.4	451.541	1.27458	0.02 ± 0.001 ^b^	0.01 ± 0.001 ^b^	0.23 ± 0.051 ^a^	0.01 ± 0.001 ^b^
2-Methylbutyl acetate	130.2	1110.1	492.504	1.72463	0.04 ± 0.003 ^c^	0.07 ± 0.004 ^b^	0.13 ± 0.01 ^a^	0.07 ± 0.002 ^b^
Isopropyl isothiocyanate-M	101.2	1134.6	535.618	1.09185	0.96 ± 0.133 ^b^	3.99 ± 0.028 ^a^	0.88 ± 0.013 ^b^	0.19 ± 0.019 ^c^
Isopropyl isothiocyanate-D	101.2	1134.6	535.618	1.09185	0.19 ± 0.007 ^d^	2.20 ± 0.024 ^a^	0.37 ± 0.032 ^b^	0.28 ± 0.010 ^c^
Isoamyl acetate-M	130.2	1138.2	542.35	1.30144	0.14 ± 0.014 ^d^	0.34 ± 0.02 ^b^	0.40 ± 0.027 ^b^	0.84 ± 0.011 ^a^
Isoamyl acetate-D	130.2	1138.2	542.35	1.30144	0.03 ± 0.004 ^c^	0.14 ± 0.011 ^b^	0.20 ± 0.039 ^b^	1.36 ± 0.10 ^a^
Ethyl pentanoate	130.2	1163.2	590.92	1.2857	0.27 ± 0.009 ^c^	0.20 ± 0.008 ^d^	0.97 ± 0.026 ^b^	2.75 ± 0.020 ^a^
Ethyl hexanoate-M	144.2	1247	729.499	1.34328	0.01 ± 0.001 ^b^	0.02 ± 0.001 ^b^	0.15 ± 0.012 ^a^	0.02 ± 0.000 ^b^
Ethyl hexanoate-D	144.2	1247	729.499	1.34328	0.02 ± 0.002 ^b^	0.01 ± 0.001 ^bc^	0.03 ± 0.002 ^a^	0.01 ± 0.001 ^c^
cis-3-Hexenyl acetate	142.2	1331.7	870.734	1.32866	0.02 ± 0.003 ^a^	0.02 ± 0.003 ^a^	0.02 ± 0.001 ^a^	0.01 ± 0.002 ^a^
Ethyl lactate-M	118.1	1362.8	931.32	1.15595	0.06 ± 0.004 ^c^	0.07 ± 0.004 ^b^	1.00 ± 0.006 ^a^	0.05 ± 0.004 ^c^
Ethyl lactate-D	118.1	1362.8	931.32	1.15595	0.03 ± 0.003 ^b^	0.03 ± 0.004 ^b^	0.25 ± 0.006 ^a^	0.03 ± 0.000 ^b^
Ethyl heptanoate	158.2	1366.6	939.048	1.42191	0.05 ± 0.003 ^b^	0.03 ± 0.003 ^c^	0.04 ± 0.001 ^c^	0.16 ± 0.005 ^a^
Ethyl octanoate	172.3	1455.7	1138.39	1.48435	0.05 ± 0.006 ^a^	0.05 ± 0.004 ^a^	0.05 ± 0.005 ^ab^	0.04 ± 0.003 ^b^
gamma-Butyrolactone	86.1	1714.4	1990.701	1.09373	1.71 ± 0.082 ^a^	0.72 ± 0.015 ^c^	0.61 ± 0.010 ^d^	1.18 ± 0.007 ^b^
Total					5.82 ± 0.242 ^d^	11.02 ± 0.303 ^c^	13.28 ± 0.598 ^b^	14.09 ± 0.388 ^a^
Ketones (19)								
Acetone	58.1	836	248.138	1.11374	7.04 ± 0.181 ^a^	7.10 ± 0.170 ^a^	6.42 ± 0.037 ^b^	4.73 ± 0.116 ^c^
2-Butanone	72.1	917.3	296.352	1.24426	3.71 ± 0.040 ^a^	3.42 ± 0.064 ^b^	2.79 ± 0.007 ^c^	2.87 ± 0.095 ^c^
2-Pentanone	86.1	1000.9	356.942	1.36668	0.24 ± 0.037 ^b^	0.22 ± 0.021 ^b^	0.19 ± 0.009 ^b^	0.32 ± 0.040 ^a^
4-Methyl-2-pentanone	100.2	1027.9	385.85	1.18531	0.19 ± 0.030 ^a^	0.14 ± 0.010 ^b^	0.11 ± 0.005 ^b^	0.18 ± 0.010 ^a^
1-Penten-3-one	84.1	1044.6	404.834	1.08174	0.05 ± 0.004 ^b^	0.04 ± 0.004 ^c^	0.08 ± 0.002 ^a^	0.03 ± 0.002 ^d^
2,3-Pentanedione	100.1	1066.5	431.266	1.20009	1.15 ± 0.073 ^a^	0.88 ± 0.088 ^b^	0.48 ± 0.042 ^c^	0.19 ± 0.007 ^d^
2-Hexanone	100.2	1101.6	478.392	1.19714	0.07 ± 0.002 ^a^	0.06 ± 0.005 ^b^	0.03 ± 0.001 ^d^	0.06 ± 0.003 ^c^
3-Penten-2-one-M	84.1	1117	504.36	1.0948	1.05 ± 0.017 ^a^	0.85 ± 0.021 ^b^	0.59 ± 0.020 ^c^	0.44 ± 0.022 ^d^
3-Penten-2-one-D	84.1	1117	504.36	1.0948	0.57 ± 0.063 ^b^	0.43 ± 0.029 ^c^	0.60 ± 0.016 ^b^	0.77 ± 0.026 ^a^
4-Heptanone-M	114.2	1170.5	605.731	1.24066	0.41 ± 0.108 ^a^	0.26 ± 0.013 ^b^	0.16 ± 0.002 ^c^	0.15 ± 0.022 ^c^
4-Heptanone-D	114.2	1170.5	605.731	1.24066	0.08 ± 0.030 ^b^	0.04 ± 0.002 ^c^	0.04 ± 0.001 ^c^	0.12 ± 0.013 ^a^
2-Heptanone	114.2	1196.3	657.814	1.26308	0.11 ± 0.005 ^a^	0.08 ± 0.005 ^c^	0.06 ± 0.006 ^d^	0.09 ± 0.004 ^b^
4-Hexen-3-one	98.1	1200.3	663.297	1.1127	0.09 ± 0.004 ^a^	0.06 ± 0.002 ^b^	0.04 ± 0.002 ^d^	0.04 ± 0.001 ^c^
3-Octanone	128.2	1239.3	718.122	1.30231	0.04 ± 0.009 ^b^	0.03 ± 0.002 ^bc^	0.03 ± 0.000 ^c^	0.09 ± 0.006 ^a^
3-Hydroxy-2-butanone-M	98.1	1301.3	815.436	1.16314	0.97 ± 0.026 ^b^	0.99 ± 0.012 ^b^	0.97 ± 0.020 ^b^	1.22 ± 0.013 ^a^
3-Hydroxy-2-butanone-D	88.1	1301.3	815.436	1.09028	0.20 ± 0.013 ^c^	0.21 ± 0.008 ^c^	0.49 ± 0.012 ^b^	0.52 ± 0.016 ^a^
Cyclohexanone	88.1	1301.3	815.436	1.09028	0.11 ± 0.008 ^c^	0.13 ± 0.007 ^b^	0.18 ± 0.003 ^a^	0.12 ± 0.009 ^bc^
1-Hydroxy-2-propanone-M	74.1	1316.6	842.849	1.09589	3.48 ± 0.086 ^a^	2.91 ± 0.023 ^b^	1.32 ± 0.045 ^d^	1.79 ± 0.001 ^c^
1-Hydroxy-2-propanone-D	74.1	1316.6	842.849	1.09589	2.66 ± 0.111 ^a^	1.66 ± 0.047 ^b^	0.44 ± 0.021 ^d^	0.63 ± 0.011 ^c^
6-Methyl-5-hepten-2-one	126.2	1349.8	905.563	1.17761	0.04 ± 0.006 ^bc^	0.05 ± 0.008 ^b^	0.04 ± 0.001 ^c^	0.16 ± 0.004 ^a^
2-Acetylfuran	110.1	1544.3	1378.468	1.13048	0.54 ± 0.013 ^a^	0.14 ± 0.005 ^c^	0.13 ± 0.004 ^c^	0.42 ± 0.040 ^b^
Acetophenone	120.2	1817.9	2489.778	1.17898	0.15 ± 0.019 ^c^	0.23 ± 0.009 ^b^	0.26 ± 0.014 ^a^	0.13 ± 0.007 ^c^
Carvone	150.2	1889.8	2908.024	1.31286	0.18 ± 0.005 ^a^	0.15 ± 0.009 ^b^	0.12 ± 0.005 ^c^	0.12 ± 0.008 ^c^
Total					23.1 ± 0.181 ^a^	20.09 ± 0.378 ^b^	15.54 ± 0.114 ^c^	15.16 ± 0.210 ^c^
Alcohols (17)								
Ethanol	46.1	944.9	314.734	1.13563	7.23 ± 0.388 ^c^	10.43 ± 0.161 ^b^	17.14 ± 0.35 ^a^	5.14 ± 0.288 ^d^
2-Butanol-M	74.1	1041.2	400.917	1.15331	0.32 ± 0.006 ^c^	0.28 ± 0.004 ^d^	0.55 ± 0.011 ^a^	0.44 ± 0.009 ^b^
2-Butanol-D	74.1	1041.2	400.917	1.15331	0.09 ± 0.007 ^b^	0.10 ± 0.001 ^b^	0.28 ± 0.002 ^a^	0.30 ± 0.017 ^a^
1-Propanol-M	60.1	1057.2	419.901	1.11458	0.58 ± 0.056 ^b^	0.91 ± 0.015 ^a^	0.50 ± 0.023 ^c^	0.48 ± 0.019 ^c^
1-Propanol-D	60.1	1057.2	419.901	1.11458	0.12 ± 0.007 ^d^	0.17 ± 0.005 ^c^	0.83 ± 0.024 ^b^	0.88 ± 0.009 ^a^
2-Methyl-1-propanol-M	74.1	1111.4	494.743	1.17746	1.57 ± 0.089 ^b^	2.03 ± 0.007 ^a^	1.07 ± 0.014 ^c^	1.11 ± 0.090 ^c^
2-Methyl-1-propanol-D	74.1	1111.4	494.743	1.17746	0.45 ± 0.031 ^d^	1.44 ± 0.031 ^c^	2.69 ± 0.028 ^b^	3.5 ± 0.024 ^a^
1-Butanol-M	74.1	1162.3	588.996	1.18533	1.03 ± 0.081 ^a^	1.13 ± 0.085 ^a^	1.09 ± 0.008 ^a^	0.41 ± 0.029 ^b^
1-Butanol-D	74.1	1162.3	588.996	1.18533	0.13 ± 0.025 ^c^	0.23 ± 0.037 ^b^	0.92 ± 0.015 ^a^	0.11 ± 0.002 ^c^
3-Methyl-1-butanol-M	88.1	1223.1	694.821	1.2444	1.26 ± 0.061 ^b^	1.61 ± 0.008 ^a^	1.19 ± 0.011 ^c^	1.13 ± 0.030 ^c^
3-Methyl-1-butanol-D	88.1	1223.1	694.821	1.2444	0.59 ± 0.065 ^d^	1.65 ± 0.027 ^c^	2.49 ± 0.023 ^b^	3.61 ± 0.024 ^a^
3-Methyl-3-buten-1-ol	86.1	1266.5	759.065	1.17761	0.04 ± 0.002 ^b^	0.04 ± 0.001 ^b^	0.02 ± 0.003 ^c^	0.05 ± 0.001 ^a^
1-Pentanol-M	88.1	1268	761.297	1.2556	0.34 ± 0.013 ^b^	0.44 ± 0.015 ^a^	0.14 ± 0.007 ^d^	0.25 ± 0.005 ^c^
1-Pentanol-D	88.1	1268	761.297	1.2556	0.04 ± 0.005 ^b^	0.06 ± 0.005 ^a^	0.02 ± 0.001 ^d^	0.03 ± 0.004 ^c^
1-Hexanol	102.2	1373.7	953.405	1.33276	0.35 ± 0.012 ^a^	0.07 ± 0.002 ^d^	0.09 ± 0.002 ^c^	0.18 ± 0.007 ^b^
(E)-3-Hexen-1-ol	100.2	1389.2	985.898	1.22993	0.02 ± 0.001 ^c^	0.03 ± 0.004 ^a^	0.02 ± 0.001 ^b^	0.01 ± 0.002 ^d^
(E)-2-Hexenol	100.2	1395.1	998.58	1.32876	0.03 ± 0.003 ^c^	0.14 ± 0.005 ^a^	0.07 ± 0.012 ^b^	0.03 ± 0.014 ^c^
(Z)-3-Hexenol	100.2	1405.8	1022	1.25723	0.04 ± 0.002 ^b^	0.03 ± 0.002 ^b^	0.04 ± 0.003 ^b^	0.06 ± 0.005 ^a^
Linalool oxide	170.3	1443.9	1109.719	1.23909	0.04 ± 0.004 ^b^	0.11 ± 0.009 ^a^	0.03 ± 0.001 ^c^	0.04 ± 0.002 ^bc^
1-Octen-3-ol	128.2	1487.7	1219.784	1.16307	0.04 ± 0.003 ^c^	0.06 ± 0.002 ^b^	0.04 ± 0.003 ^c^	0.15 ± 0.012 ^a^
2-Ethyl-1-hexanol	130.2	1542.1	1371.811	1.43037	0.09 ± 0.010 ^a^	0.09 ± 0.006 ^a^	0.05 ± 0.011 ^b^	0.06 ± 0.004 ^b^
Linalool	154.3	1639.9	1694.692	1.2236	0.34 ± 0.028 ^c^	0.38 ± 0.011 ^b^	0.43 ± 0.017 ^a^	0.42 ± 0.023 ^a^
alpha-Terpineol	154.3	1829.7	2553.831	1.23986	0.15 ± 0.014 ^c^	0.90 ± 0.071 ^a^	0.37 ± 0.059 ^b^	0.14 ± 0.025 ^c^
Total					14.86 ± 0.637 ^d^	22.33 ± 0.168 ^b^	30.07 ± 0.484 ^a^	18.51 ± 0.481 ^c^
Aldehydes (18)								
Acetaldehyde	44.1	762.5	211.375	1.02953	2.39 ± 0.176 ^a^	2.06 ± 0.058 ^b^	1.76 ± 0.048 ^c^	1.84 ± 0.024 ^c^
Propanal	58.1	816.1	237.591	1.14237	1.76 ± 0.056 ^a^	1.68 ± 0.076 ^ab^	1.42 ± 0.014 ^c^	1.62 ± 0.013 ^b^
2-Methylpropanal	72.1	826.4	243.015	1.28047	0.64 ± 0.013 ^b^	0.55 ± 0.014 ^c^	0.47 ± 0.005 ^d^	0.76 ± 0.026 ^a^
2-Propenal	56.1	880	273.149	1.06574	0.15 ± 0.006 ^a^	0.16 ± 0.015 ^a^	0.11 ± 0.003 ^b^	0.10 ± 0.014 ^b^
3-Methylbutanal	86.1	930.6	305.091	1.40594	4.24 ± 0.041 ^a^	3.89 ± 0.027 ^b^	2.55 ± 0.005 ^d^	3.57 ± 0.078 ^c^
Pentanal	86.1	1003.8	359.935	1.41773	0.67 ± 0.137 ^a^	0.63 ± 0.054 ^a^	0.13 ± 0.011 ^b^	0.60 ± 0.041 ^a^
Hexanal-M	100.2	1103.1	480.797	1.30243	0.66 ± 0.088 ^a^	0.7 ± 0.020 ^a^	0.34 ± 0.009 ^c^	0.49 ± 0.008 ^b^
Hexanal-D	100.2	1103.1	480.797	1.30243	0.09 ± 0.023 ^a^	0.1 ± 0.003 ^a^	0.03 ± 0.002 ^c^	0.06 ± 0.006 ^b^
(E)-2-Pentenal-M	84.1	1151.4	567.357	1.1076	0.68 ± 0.032 ^a^	0.62 ± 0.037 ^b^	0.26 ± 0.006 ^c^	0.08 ± 0.002 ^d^
(E)-2-Pentenal-D	84.1	1151.4	567.357	1.1076	0.25 ± 0.03 ^a^	0.21 ± 0.026 ^b^	0.24 ± 0.005 ^ab^	0.07 ± 0.003 ^c^
Heptanal	114.2	1198.8	661.241	1.36488	0.06 ± 0.006 ^b^	0.07 ± 0.003 ^a^	0.03 ± 0.004 ^c^	0.03 ± 0.001 ^c^
3-Methyl-2-butenal-M	84.1	1217.3	686.598	1.09215	0.49 ± 0.018 ^a^	0.17 ± 0.012 ^c^	0.35 ± 0.018 ^b^	0.35 ± 0.022 ^b^
3-Methyl-2-butenal-D	84.1	1217.3	686.598	1.09215	0.12 ± 0.009 ^c^	0.07 ± 0.004 ^d^	0.15 ± 0.017 ^b^	0.27 ± 0.022 ^a^
(E)-2-Heptenal	112.2	1334.3	875.744	1.25747	0.06 ± 0.006 ^b^	0.06 ± 0.006 ^b^	0.10 ± 0.003 ^a^	0.06 ± 0.003 ^b^
Nonanal	142.2	1408.9	1028.773	1.50194	0.12 ± 0.002 ^a^	0.11 ± 0.006 ^b^	0.07 ± 0.004 ^d^	0.07 ± 0.004 ^c^
(E)-2-Octenal	126.2	1441.1	1102.937	1.33782	0.05 ± 0.004 ^ab^	0.04 ± 0.003 ^b^	0.06 ± 0.014 ^a^	0.04 ± 0.014 ^b^
Citronellal	154.3	1488.2	1221.009	1.22339	0.06 ± 0.003 ^bc^	0.08 ± 0.006 ^b^	0.05 ± 0.005 ^c^	0.28 ± 0.024 ^a^
Furfural	96.1	1497.3	1245.303	1.3369	0.10 ± 0.010 ^b^	0.08 ± 0.008 ^b^	0.11 ± 0.032 ^b^	1.30 ± 0.178 ^a^
Benzaldehyde	106.1	1554.9	1410.351	1.15785	0.46 ± 0.012 ^b^	0.4 ± 0.013 ^c^	0.49 ± 0.016 ^b^	0.61 ± 0.014 ^a^
Salicylic aldehyde	122.1	1735.8	2084.855	1.13761	0.23 ± 0.010 ^b^	0.19 ± 0.016 ^c^	0.17 ± 0.009 ^c^	0.37 ± 0.04 ^a^
Phenylacetaldehyde	120.2	1770.3	2246.508	1.26985	0.71 ± 0.098 ^b^	0.53 ± 0.022 ^c^	0.50 ± 0.033 ^c^	1.47 ± 0.097 ^a^
Total					13.98 ± 0.209 ^a^	12.37 ± 0.137 ^b^	9.38 ± 0.081 ^c^	14.03 ± 0.153 ^a^
Hydrocarbons (8)								
alpha-Pinene	136.2	1031.2	389.466	1.28974	0.04 ± 0.002 ^d^	0.09 ± 0.007 ^c^	0.18 ± 0.009 ^a^	0.13 ± 0.011 ^b^
beta-Pinene	136.2	1124.8	518.021	1.21216	0.04 ± 0.002 ^c^	0.05 ± 0.001 ^b^	0.09 ± 0.006 ^a^	0.09 ± 0.006 ^a^
p-Xylene	106.2	1147.6	560.143	1.07808	0.25 ± 0.010 ^a^	0.24 ± 0.003 ^a^	0.12 ± 0.003 ^b^	0.07 ± 0.003 ^c^
o-Xylene	106.2	1192.2	652.332	1.09215	0.18 ± 0.020 ^a^	0.18 ± 0.001 ^a^	0.10 ± 0.001 ^b^	0.09 ± 0.004 ^b^
alpha-Terpinene	136.2	1193.2	653.703	1.22665	0.08 ± 0.011 ^b^	0.06 ± 0.003 ^bc^	0.05 ± 0.006 ^c^	0.32 ± 0.025 ^a^
Limonene	136.2	1207.3	672.765	1.21574	0.02 ± 0.002 ^b^	0.07 ± 0.001 ^a^	0.02 ± 0.003 ^b^	0.02 ± 0.001 ^b^
gamma-Terpinene	136.2	1255.9	742.793	1.21637	0.03 ± 0.001 ^b^	0.02 ± 0.002 ^bc^	0.15 ± 0.010 ^a^	0.01 ± 0.001 ^c^
Terpinolene	136.2	1281.1	781.856	1.20423	0.54 ± 0.012 ^a^	0.21 ± 0.010 ^b^	0.07 ± 0.003 ^d^	0.16 ± 0.013 ^c^
Total					1.17 ± 0.012 ^a^	0.92 ± 0.021 ^b^	0.76 ± 0.031 ^c^	0.89 ± 0.026 ^b^
Acids (5)								
Acetic acid-M	60.1	1505.2	1266.879	1.06157	16.06 ± 0.393 ^a^	13.57 ± 0.176 ^b^	13.65 ± 0.07 ^b^	11.09 ± 0.278 ^c^
Acetic acid-D	60.1	1505.2	1266.879	1.06157	7.21 ± 0.488 ^a^	3.91 ± 0.110 ^c^	4.23 ± 0.050 ^bc^	4.57 ± 0.097 ^b^
2-Methylpropanoic acid-M	88.1	1637.8	1687.06	1.15633	3.31 ± 0.236 ^b^	2.64 ± 0.038 ^c^	2.60 ± 0.035 ^c^	7.90 ± 0.308 ^a^
2-Methylpropanoic acid-D	88.1	1637.8	1687.06	1.15633	0.26 ± 0.021 ^b^	0.21 ± 0.012 ^b^	0.19 ± 0.013 ^b^	1.20 ± 0.142 ^a^
Propanoic acid	74.1	1640.1	1695.379	1.12238	2.05 ± 0.054 ^a^	0.99 ± 0.048 ^c^	0.73 ± 0.016 ^d^	1.35 ± 0.054 ^b^
Butanoic acid	88.1	1713.4	1986.541	1.17542	0.50 ± 0.045 ^d^	0.62 ± 0.019 ^c^	0.92 ± 0.041 ^b^	1.51 ± 0.079 ^a^
Pentanoic acid	102.1	1910.3	3039.964	1.23887	0.98 ± 0.087 ^c^	0.65 ± 0.081 ^d^	1.22 ± 0.023 ^b^	2.01 ± 0.122 ^a^
Total					30.36 ± 0.744 ^a^	22.59 ± 0.250 ^c^	23.54 ± 0.111 ^b^	29.62 ± 0.300 ^a^
Others (23)								
Dimethyl sulfide	62.1	792.7	225.753	0.95459	0.98 ± 0.150 ^c^	0.83 ± 0.081 ^c^	1.26 ± 0.071 ^b^	1.57 ± 0.120 ^a^
2-Methylfuran	82.1	884	275.56	0.97226	0.08 ± 0.010 ^ab^	0.09 ± 0.018 ^a^	0.09 ± 0.012 ^a^	0.06 ± 0.008 ^b^
Acetal	118.2	905.5	288.819	1.02363	1.94 ± 0.094 ^a^	1.13 ± 0.033 ^b^	0.52 ± 0.030 ^c^	0.38 ± 0.059 ^d^
2-Ethylfuran	96.1	1004.6	360.718	1.31692	0.24 ± 0.010 ^c^	0.55 ± 0.034 ^a^	0.39 ± 0.026 ^b^	0.20 ± 0.046 ^d^
Acrylonitrile	53.1	1033	391.575	1.04889	2.06 ± 0.097 ^a^	1.77 ± 0.031 ^b^	0.95 ± 0.026 ^c^	0.64 ± 0.116 ^d^
Diallyl sulfide	114.2	1165.5	595.438	1.1204	0.06 ± 0.002 ^c^	0.68 ± 0.045 ^a^	0.18 ± 0.006 ^b^	0.03 ± 0.002 ^c^
1,8-Cineole-M	154.3	1214.8	683.171	1.30978	0.18 ± 0.028 ^d^	1.50 ± 0.016 ^a^	0.35 ± 0.007 ^c^	0.49 ± 0.021 ^b^
1,8-Cineole-D	154.3	1214.8	683.171	1.30978	0.04 ± 0.003 ^d^	0.23 ± 0.006 ^a^	0.07 ± 0.002 ^c^	0.08 ± 0.004 ^b^
Pyrazine	80.1	1229.4	703.73	1.05292	0.21 ± 0.004 ^c^	0.33 ± 0.024 ^b^	0.64 ± 0.018 ^a^	0.08 ± 0.012 ^d^
2-Pentylfuran	138.2	1246.3	728.402	1.25187	0.04 ± 0.004 ^b^	0.05 ± 0.002 ^b^	0.05 ± 0.007 ^b^	0.14 ± 0.011 ^a^
2-Methylpyrazine-M	94.1	1281.5	782.541	1.08094	1.21 ± 0.020 ^a^	0.72 ± 0.007 ^b^	0.42 ± 0.005 ^c^	0.38 ± 0.011 ^d^
2-Methylpyrazine-D	94.1	1281.5	782.541	1.08094	0.83 ± 0.049 ^a^	0.13 ± 0.004 ^b^	0.04 ± 0.003 ^c^	0.09 ± 0.008 ^b^
2,5-Dimethylpyrazine	108.1	1331.1	869.576	1.12858	0.07 ± 0.001 ^a^	0.05 ± 0.002 ^b^	0.02 ± 0.001 ^c^	0.01 ± 0.000 ^d^
2,6-Dimethylpyrazine	108.1	1339.7	886.023	1.12577	0.19 ± 0.014 ^a^	0.05 ± 0.003 ^b^	0.05 ± 0.002 ^b^	0.02 ± 0.000 ^c^
Ethylpyrazine	108.1	1354.6	914.964	1.12692	0.14 ± 0.011 ^a^	0.07 ± 0.005 ^b^	0.06 ± 0.003 ^c^	0.07 ± 0.007 ^b^
2,3-Dimethylpyrazine	108.1	1370.7	947.22	1.12358	0.07 ± 0.002 ^d^	0.08 ± 0.002 ^c^	0.10 ± 0.004 ^b^	0.17 ± 0.013 ^a^
Dipropyl disulfide	150.3	1383.6	974.026	1.25638	0.04 ± 0.002 ^bc^	0.34 ± 0.008 ^a^	0.05 ± 0.007 ^b^	0.03 ± 0.004 ^c^
3-Ethylpyridine	107.2	1389.4	986.446	1.12353	0.48 ± 0.022 ^a^	0.30 ± 0.008 ^b^	0.13 ± 0.005 ^c^	0.13 ± 0.007 ^c^
2,3,5-Trimethylpyrazine	122.2	1409.5	1030.233	1.17033	0.03 ± 0.006 ^b^	0.03 ± 0.004 ^b^	0.03 ± 0.002 ^b^	0.05 ± 0.003 ^a^
2,6-Dimethyl-3-ethylpyrazine	136.2	1473.3	1182.384	1.23338	0.03 ± 0.007 ^a^	0.03 ± 0.003 ^a^	0.03 ± 0.006 ^a^	0.04 ± 0.002 ^a^
Methional-M	104.2	1478.3	1195.143	1.09197	0.24 ± 0.016 ^d^	0.32 ± 0.013 ^c^	0.51 ± 0.019 ^b^	0.86 ± 0.02 ^a^
Methional-D	104.2	1478.3	1195.143	1.09197	0.04 ± 0.001 ^c^	0.04 ± 0.003 ^c^	0.06 ± 0.006 ^b^	0.15 ± 0.009 ^a^
Diallyl disulfide	146.3	1520.7	1309.958	1.20671	0.08 ± 0.016 ^a^	0.09 ± 0.009 ^a^	0.04 ± 0.002 ^b^	0.03 ± 0.002 ^b^
2-Isobutyl-3-methoxypyrazine-M	166.2	1547.7	1388.648	1.29017	0.23 ± 0.021 ^ab^	0.25 ± 0.024 ^a^	0.23 ± 0.008 ^ab^	0.20 ± 0.025 ^b^
2-Isobutyl-3-methoxypyrazine-D	166.2	1547.7	1388.648	1.29017	0.09 ± 0.008 ^a^	0.09 ± 0.007 ^a^	0.07 ± 0.002 ^b^	0.07 ± 0.000 ^b^
2-Acetyl-3-methylpyrazine	136.2	1694.6	1907.511	1.17118	0.37 ± 0.018 ^c^	0.42 ± 0.017 ^c^	0.65 ± 0.016 ^b^	1.20 ± 0.103 ^a^
3-(Methylthio)-1-propanol	106.2	1803.4	2412.623	1.10649	0.70 ± 0.035 ^a^	0.49 ± 0.083 ^b^	0.44 ± 0.067 ^b^	0.51 ± 0.016 ^b^
Total					10.68 ± 0.193 ^a^	10.66 ± 0.091 ^a^	7.42 ± 0.108 ^b^	7.69 ± 0.155 ^b^

Abbreviations: MW—molecular weight; RI—retention index; RT—retention time; Dt—drift time; M—monomer; D—dimer. Values marked with lower case letters in the same line were significantly different (*p* < 0.05).

## Data Availability

The original contributions presented in this study are included in the article. Further inquiries can be directed to the corresponding author.
